# Supplementary material for: Cross-sectional associations between body mass index, waist circumference, and multimorbidity: Pró-Saúde study
Source: PeerJ. 2023 Feb 6;11:e14744. doi: 10.7717/peerj.14744 (PMC9910183; doi:10.7717/peerj.14744)
Supplement: Supplemental Information 1 [file peerj-11-14744-s001.docx]

| Supplemmentary Table – Odds ratio unadjusted and 95% confidence interval (CI) of the association between BMI and WC and the multimorbidity categories . | | | | | | |  |
| --- | --- | --- | --- | --- | --- | --- | --- |
|  |  |  |  |  |  |  |  |
| **BMI** | | | |  | | |  |
| **Number of morbidities** | OR adjusted (IC95%) | | | OR adjusted (IC95%) | | |  |
|  | Total | Female | Male | Total | Female | Male |  |
| **2** | 1.84 | 1.73 | 1.90 | 2.43 | 2.94 | 3.20 |  |
|  | (1.45-2.23) | (1.24-2.57) | (1.24-2.57) | (1.90-2.96) | (2.24-3.63) | (2.30-4.10) |  |
| **3** | 2.81 | 2.68 | 2.95 | 3.40 | 3.89 | 4.26 |  |
|  | (2.41-3,21) | (2.18-3.17) | (2.28-3.63) | (2.86-3.93) | (3.18-4.59) | (3.34-5.17) |  |
| **More than 4** | 3.70 | 3.59 | 3.84 | 4.27 | 4.81 | 5.14 |  |
|  | (3.29-4.10) | (3.08-4.53) | (3.14-4.53) | (3.72-4.82) | (4.09-5.53) | (4.21-6.07) |  |

^p value<0,001 for all variables^
